# Clinically Suspected Acute Myocarditis in the First Trimester of Twin Gestation: A Case Report

**DOI:** 10.7759/cureus.50851

**Published:** 2023-12-20

**Authors:** Herson S Flores-Sanga, Dafne Salas-Cuadros, Jose Luis Saucedo-Chinchay, Jose Arriola-Montenegro, Javier Torres-Valencia, Juan Manuel Muñoz-Moreno

**Affiliations:** 1 Department of Cardiology, Hospital Nacional Carlos Alberto Seguin Escobedo, Arequipa, PER; 2 Victor Alzamora Castro Postgraduate School, Universidad Peruana Cayetano Heredia, Lima, PER; 3 Department of Cardiology, Hospital Nacional Edgardo Rebagliati Martins, Lima, PER; 4 Department of Internal Medicine, University of Minnesota, Minneapolis, USA; 5 Department of Cardiology, Instituto Nacional Cardiovascular, Lima, PER

**Keywords:** diseases in twins, cardiac magnetic resonance imaging, twin pregnancy, myocardium, myocarditis

## Abstract

Acute myocarditis (AM) in early pregnancy is a rare disease. Its clinical presentation varies from asymptomatic disease to cardiogenic shock and death. A 28-year-old woman, 12 weeks primigravida of a dichorionic and diamniotic pregnancy, was admitted for hyperemesis gravidarum, associated with a common cold-like condition. During hospitalization, she developed new-onset sinus tachycardia and dyspnea. An electrocardiogram revealed sinus tachycardia and diffuse ST-segment elevation. Laboratory tests showed elevated levels of troponin and pro-B-type natriuretic peptide. Pelvic obstetric ultrasound and chest X-ray were normal. Speckle-tracking echocardiography showed mild apical hypokinesia with preserved left ventricular ejection fraction. In view of these findings, AM was suspected, and cardiac magnetic resonance imaging was highly suggestive of AM. The patient had a favorable recovery without cardiovascular or obstetric complications.

## Introduction

Pregnant women often experience nausea and vomiting during the first trimester of pregnancy, usually attributed to hyperemesis gravidarum as the most common diagnosis, but it is important to consider the possibility of other serious underlying causes, such as acute myocarditis (AM) [1]. AM during the first trimester of pregnancy is rare and its true incidence is unknown, as it typically occurs in the third trimester associated with peripartum cardiomyopathy, with viral etiology being the most common cause [2,3].

In this report, we present the case of a 28-year-old woman, primigravida of 12 weeks of twin gestation, who presented clinical features suspicious of AM, supported by the findings in cardiac magnetic resonance imaging (MRI).

## Case presentation

A 28-year-old woman, pregnant for the first time, presented with a five-day history of fluid intolerance at 12 weeks' gestation in a dichorionic diamniotic pregnancy. Since eight weeks of gestation, she had been experiencing severe vomiting, resulting in a significant weight loss of approximately 10 kg. She also had a one-week history of common cold-like symptoms. On admission, her temperature was 36.8°C, her pulse rate was 110 beats/min, and her blood pressure was 100/60 mmHg. Laboratory tests revealed normal levels of hemoglobin (13.1 g/dL NV:12-16), leukocytes (9.81×103/uL NV:4-10.5), and platelets. An electrolyte screen showed moderate hypokalemia (2.97 mmol/L NV: 3.5-5) with normal levels of sodium (139 mmol/L NV: 135-145) and chloride (102 mmol/L NV: 102-109). In addition, transaminases were elevated (aspartate aminotransferase of 326 U/L NV:8-48 and alanine aminotransferase of 115 U/L NV:7-55), with normal creatinine and albumin levels. The molecular PCR test for SARS-CoV-2 was negative. Obstetric pelvic ultrasound and chest x-ray were unremarkable. 

**Figure 1:**
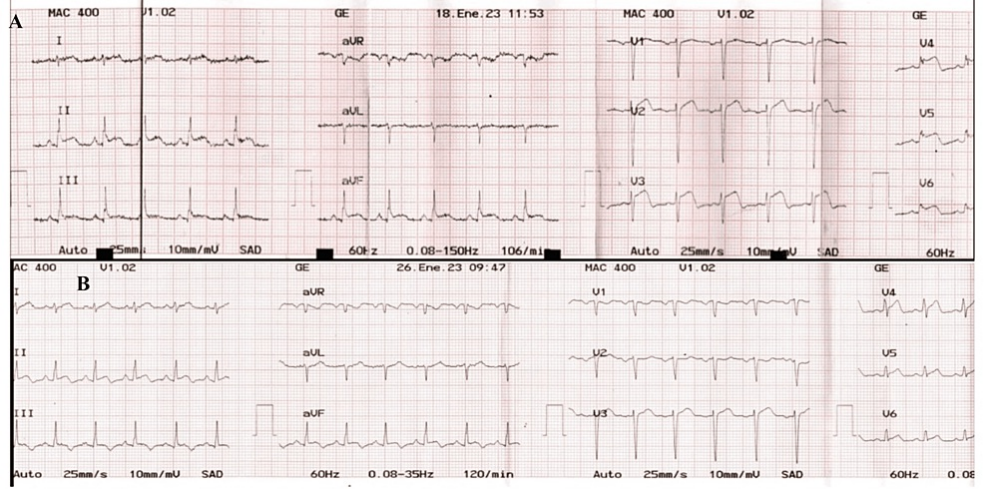
Initial and follow-up electrocardiogram (A) Electrocardiogram showing sinus tachycardia (107 beats/min) and ST elevation in leads I, II, III, AVF, and V2-6. (B) A follow-up electrocardiogram showed a regression of abnormalities in the ST segment.

On the third day of the obstetric hospitalization, the patient continued to have sinus tachycardia and developed dyspnea, for which she was evaluated by the cardiology service. The physical examination was normal. The 12-lead electrocardiogram (ECG) showed sinus tachycardia, and diffuse ST-segment elevation in leads I, II, III, AVF, and V2-6 (Figure 1A). The pre-discharge ECG showed regression of the ST segment changes (Figure 1B). The patient did not require any treatment because she remained hemodynamically stable and had a favorable outcome with no cardiovascular or obstetric complications and was discharged at the end of delivery.

**Figure 2:**
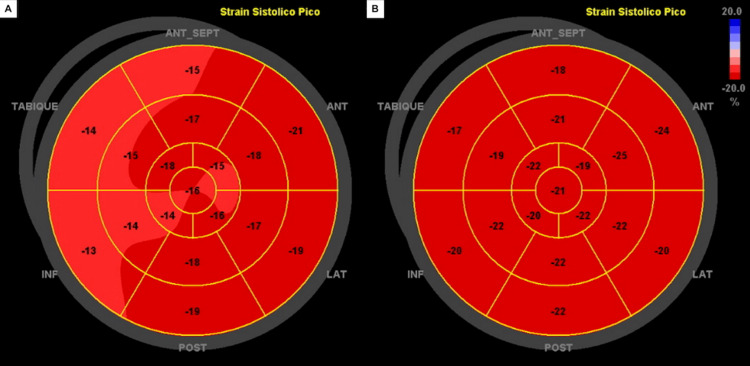
Bull's eye plot of the speckle-tracking analysis (A) The global longitudinal strain was impaired by −15.9% at presentation. (B) At the three-month follow-up, the strain analysis was normal.

Further laboratory studies revealed elevated troponin I (15.85 ng/mL NV: 0.00-0.015) and pro-B-type natriuretic peptide (584 pg/mL NV:<125). Speckle-tracking echocardiography (STE) showed mild apical hypokinesis with a left ventricular ejection fraction (LVEF) of 58%. The global left ventricular longitudinal strain was reduced (-15.9%) (Figure 2A). At three months of outpatient follow-up, the patient is asymptomatic and the STE showed normal motility of all myocardial segments (Figure 2B), demonstrating a complete reversal of the changes observed during hospitalization.

Based on these findings, AM was clinically suspected and cardiac MRI was performed without gadolinium administration due to the patient's pregnancy.

**Figure 3:**
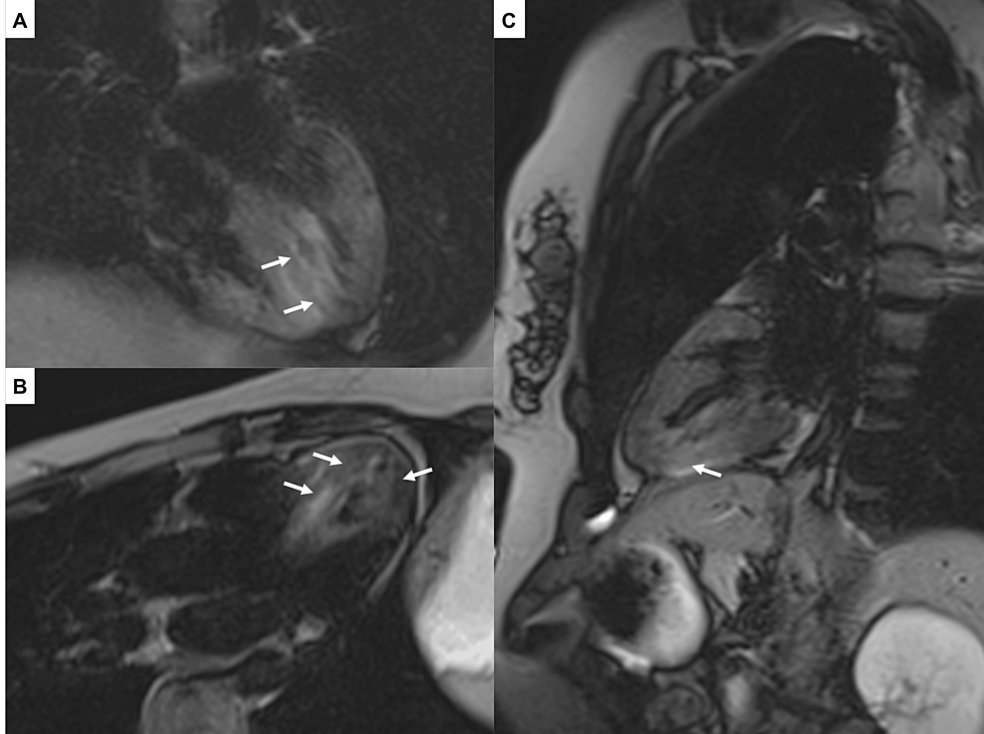
Cardiac magnetic resonance imaging Four-chamber (A), three-chamber (B), and two-chamber (C) view T2-weighted images. The white arrows indicate myocardial edema in mid-septal and apical septal, inferior, and lateral segments.

Cardiac MRI revealed an LVEF of 55-60% and edema in the septal, inferior, and lateral segments, highly suggestive of AM (Figure 3).

There was also septal wall thickening and reduced motion of the apical and posterior segments of the interventricular septum. Cardiac MRI parametric mapping could not be performed due to unavailability in our hospital. Endomyocardial biopsy (EMB) was not performed because the patient was hemodynamically stable and to avoid exposing her and the fetus to radiation.

## Discussion

AM refers to a severe lymphocyte-mediated inflammatory disorder of the myocardium and is defined by established histologic, immunologic, and immunohistochemical criteria according to the 1995 World Health Organization/International Society and Federation of Cardiology Task Force [4]. It affects millions of people worldwide, especially children and young adults, both sexes and different races [5]. According to the 2019 Global Burden of the Disease report, the estimated rate of AM is 6.1 per 100,000, although AM in the first trimester of pregnancy is much rarer and its true frequency is unknown [6]. Few cases of AM in the first trimester of pregnancy have been described; most of these cases occur in the third trimester and are associated with peripartum cardiomyopathy. The clinical cases underscore the importance of vigilant and careful management of cardiac conditions during pregnancy. Gluszak and colleagues present an eight-week pregnant patient who experienced heart failure due to myocarditis. Fortunately, the patient showed a favorable outcome, emphasizing the need for early and effective medical attention [7]. In contrast, the case presented by Moore and colleagues involved a 12-week pregnant patient with lymphocytic myocarditis and dilated cardiomyopathy, unfortunately resulting in a fatal outcome [1].

AM accounts for most cases of myocarditis (65%), although the underlying cause often cannot be identified (50%) [5]. It is mainly caused by viral disease (direct cytopathic effects of cardiotropic viruses and virus-induced anticardiac immune response) and less commonly by non-infectious etiologies [8]. 

Clinical presentation includes fever, malaise, fatigue, chest pain, palpitations, dyspnea, orthopnea, or syncope [5]. Sixty percent of patients usually have a history of recent acute febrile illness, such as the common cold [5]. Early recognition is important because in some cases it can be the cause of ventricular arrhythmias and heart block or can mimic acute myocardial infarction, hemodynamic instability, and even circulatory collapse with severe left and/or right ventricular dysfunction or associated cardiac tamponade, systemic and pulmonary emboli, or sudden cardiac death [3].

The ECG, usually the first ancillary study, is often abnormal in 85% of patients [9], and most commonly shows ST-segment elevation, often in the inferior and lateral leads, as described in our patient [10]. Other ECG findings include bradycardia, tachycardia, QRS > 120 ms, atrioventricular block, and ventricular arrhythmias [11,12]. Laboratory tests include cardiac biomarkers such as troponin and brain natriuretic peptide, which are usually elevated in more than half of patients, as in our case [5].

STE is part of the standard evaluation for suspected AM; however, there is a wide range of possible findings in AM [13]. Even when the LVEF is normal, the presence of segmental wall motion abnormalities, increased cardiac wall thickness, and myocardial echogenicity, as observed in our patient, may suggest AM [13].

Cardiac MRI provides non-invasive tissue characterization of the myocardium, and the updated 2018 Lake Louise criteria (LLC) are used to establish the diagnosis of AM [14,15]. Definitive diagnosis requires compliance with the two proposed LLC, which include myocardial edema (T2-weighted images (T2w-STIR) or T2 mapping) and non-ischemic myocardial injury (late gadolinium enhancement (LGE) or T1 mapping or extracellular volume) [14,15]. The American College of Radiology, the European Society of Urogenital Radiology, and the Royal College of Radiology recommend avoidance of the use of gadolinium during pregnancy and limitation of its use only when the potential benefits clearly outweigh the potential risks to the pregnant woman or fetus [16]. In our patient, the criteria for enhanced myocardial edema on T2w-STIR were met, and the LGE criterion could not be demonstrated because the patient was pregnant. Based on the clinical suspicion of AM, the stable hemodynamic status, and the favorable evolution, it was decided to perform MRI without contrast, as the risks to the fetus outweighed the benefits. In addition, it is important to emphasize that the presence of these two criteria increases the specificity of the diagnosis, and if only one of them is present, the diagnosis is still probable, but with less specificity [14].

EMB is the "gold standard" technique for definitive diagnosis of AM, especially in patients with hemodynamic instability [17]. EMB is also used to identify the histologic type of AM (giant cell, eosinophilic, or lymphocytic) [17]. In our case, EMB was not performed because the patient was hemodynamically stable and because it was an invasive procedure with radiation, which posed a high risk to the pregnant woman and the fetus.

## Conclusions

AM is a complex and rare disease in the first trimester of pregnancy and even more so in a twin pregnancy. Clinical suspicion is essential for the diagnosis of AM. In hemodynamically stable patients, cardiac MRI is useful for definitive diagnosis, with the limitation that the use of gadolinium is not recommended in pregnant women.

## References

[1] Moore RC, Briery CM, Rose CH, Skelton TN, Martin JN Jr (2006). Lymphocytic myocarditis presenting as nausea, vomiting, and hepatic dysfunction in the first trimester of pregnancy. Obstet Gynecol.

[2] Massengill A, Rodriguez J, Cotter T, Cotter JG (2016). 42. Infectious myocarditis in pregnancy: an unlikely cause of respiratory distress. Am J Obstet Gynecol.

[3] Shotan A, Keren A (2019). Myocarditis and pregnancy. Cardiac Problems in Pregnancy.

[4] Richardson P, McKenna W, Bristow M (1996). Report of the 1995 World Health Organization/International Society and Federation of Cardiology Task Force on the Definition and Classification of cardiomyopathies. Circulation.

[5] Al-Akchar M, Shams P, Kiel J (2024). Acute myocarditis. StatPearls [Internet].

[6] Roth GA, Mensah GA, Johnson CO (2020). Global burden of cardiovascular diseases and risk factors, 1990-2019: update from the GBD 2019 Study. J Am Coll Cardiol.

[7] Głuszak M, Borowiecka E, Borowiecka-Elwertowska A, Barcz E (2013). Acute heart failure due to myocarditis in the first trimester of pregnancy (Article in Polish). Ginekol Pol.

[8] Kyaw T, Drummond G, Bobik A, Peter K (2023). Myocarditis: causes, mechanisms, and evolving therapies. Expert Opin Ther Targets.

[9] Ammirati E, Cipriani M, Moro C (2018). Clinical presentation and outcome in a contemporary cohort of patients with acute myocarditis: multicenter Lombardy Registry. Circulation.

[10] Lampejo T, Durkin SM, Bhatt N, Guttmann O (2021). Acute myocarditis: aetiology, diagnosis and management. Clin Med (Lond).

[11] Ammirati E, Veronese G, Brambatti M (2019). Fulminant versus acute nonfulminant myocarditis in patients with left ventricular systolic dysfunction. J Am Coll Cardiol.

[12] Younis A, Matetzky S, Mulla W (2020). Epidemiology characteristics and outcome of patients with clinically diagnosed acute myocarditis. Am J Med.

[13] Ammirati E, Frigerio M, Adler ED (2020). Management of acute myocarditis and chronic inflammatory cardiomyopathy: an expert consensus document. Circ Heart Fail.

[14] Polte CL, Bobbio E, Bollano E (2022). Cardiovascular magnetic resonance in myocarditis. Diagnostics (Basel).

[15] Ferreira VM, Schulz-Menger J, Holmvang G (2018). Cardiovascular magnetic resonance in nonischemic myocardial inflammation: expert recommendations. J Am Coll Cardiol.

[16] Gatta G, Di Grezia G, Cuccurullo V (2021). MRI in pregnancy and precision medicine: a review from literature. J Pers Med.

[17] Ammirati E, Buono A, Moroni F (2022). State-of-the-art of endomyocardial biopsy on acute myocarditis and chronic inflammatory cardiomyopathy. Curr Cardiol Rep.

